# (*E*)-3-[3-(4-Bromo­phen­yl)-1-phenyl-1*H*-pyrazol-4-yl]-1-(2,4-dichloro­phen­yl)prop-2-en-1-one

**DOI:** 10.1107/S1600536811044424

**Published:** 2011-10-29

**Authors:** Hoong-Kun Fun, Ching Kheng Quah, Shridhar Malladi, Arun M. Isloor, Kammasandra N. Shivananda

**Affiliations:** a X-ray Crystallography Unit, School of Physics, Universiti Sains Malaysia, 11800 USM, Penang, Malaysia; b Medicinal Chemistry Division, Department of Chemistry, National Institute of Technology-Karnataka, Surathkal, Mangalore 575 025, India; c Schulich Faculty of Chemistry, Technion Israel Institute of Technology, Haifa 32000, Israel

## Abstract

In the title mol­ecule, C_24_H_15_BrCl_2_N_2_O, the dihedral angles betwen the pyrazole ring and its N-bonded phenyl (*A*) and C-bonded bromo­benzene (*B*) rings are 10.34 (16) and 40.95 (15)°, respectively. The dihedral angle between rings *A* and *B* is 56.89 (17)°. The title mol­ecule exists in a *trans* conformation with respect to the acyclic C=C bond. In the crystal, mol­ecules are linked into inversion dimers by pairs of C—H⋯O hydrogen bonds, generating *R* ^2^_2_(14) loops. The crystal structure is further consolidated by C—H⋯π inter­actions.

## Related literature

For a related structure and background references to pyrazoles, see: Fun *et al.* (2011 ▶). For standard bond-length data, see: Allen *et al.* (1987 ▶). For hydrogen-bond motifs, see: Bernstein *et al.* (1995 ▶).
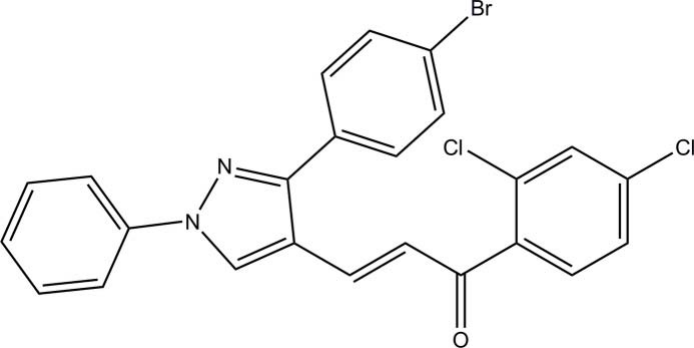


## Experimental

### 

#### Crystal data

C_24_H_15_BrCl_2_N_2_O
*M* *_r_* = 498.19
Monoclinic, 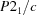

*a* = 11.4203 (14) Å
*b* = 9.9357 (13) Å
*c* = 19.656 (3) Å
β = 94.653 (3)°
*V* = 2222.9 (5) Å^3^
*Z* = 4
Mo *K*α radiation
μ = 2.11 mm^−1^
*T* = 296 K
0.38 × 0.21 × 0.11 mm

#### Data collection

Bruker SMART APEXII DUO CCD diffractometer
Absorption correction: multi-scan (*SADABS*; Bruker, 2009 ▶) *T*_min_ = 0.504, *T*_max_ = 0.803
23842 measured reflections
6480 independent reflections
2743 reflections with *I* > 2σ(*I*)
*R*_int_ = 0.056

#### Refinement

*R*[*F* ^2^ > 2σ(*F* ^2^)] = 0.052
*wR*(*F* ^2^) = 0.140
*S* = 0.98
6480 reflections
271 parameters
H-atom parameters constrained
Δρ_max_ = 0.44 e Å^−3^
Δρ_min_ = −0.52 e Å^−3^

### 

Data collection: *APEX2* (Bruker, 2009 ▶); cell refinement: *SAINT* (Bruker, 2009 ▶); data reduction: *SAINT*; program(s) used to solve structure: *SHELXTL* (Sheldrick, 2008 ▶); program(s) used to refine structure: *SHELXTL*; molecular graphics: *SHELXTL*; software used to prepare material for publication: *SHELXTL* and *PLATON* (Spek, 2009 ▶).

## Supplementary Material

Crystal structure: contains datablock(s) global, I. DOI: 10.1107/S1600536811044424/hb6462sup1.cif

Structure factors: contains datablock(s) I. DOI: 10.1107/S1600536811044424/hb6462Isup2.hkl

Supplementary material file. DOI: 10.1107/S1600536811044424/hb6462Isup3.cml

Additional supplementary materials:  crystallographic information; 3D view; checkCIF report

## Figures and Tables

**Table 1:** Hydrogen-bond geometry (Å, °) *Cg*1 is the centroid of C1–C6 phenyl ring.

*D*—H⋯*A*	*D*—H	H⋯*A*	*D*⋯*A*	*D*—H⋯*A*
C11—H11*A*⋯O1^i^	0.93	2.41	3.329 (4)	170
C15—H15*A*⋯*Cg*1^ii^	0.93	2.82	3.666 (3)	152

Symmetry codes: (i) 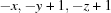; (ii) 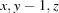.

**Fig. 1.**
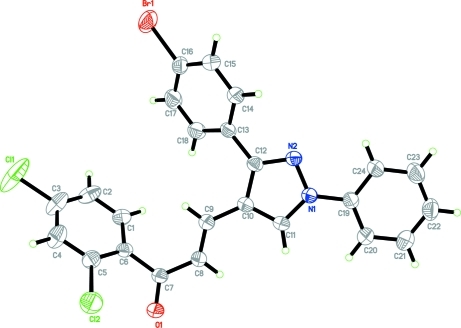
The molecular structure of the title compound showing 30% probability displacement ellipsoids for non-H atoms.

**Fig. 2.**
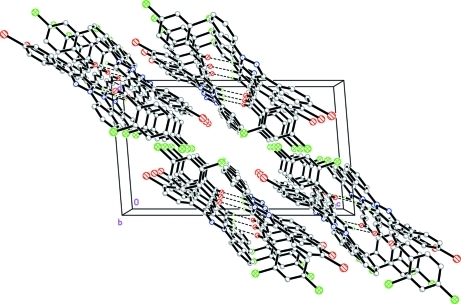
The crystal structure of the title compound, viewed along the b axis. H atoms not involved in hydrogen bonds (dashed lines) have been omitted for clarity.

## supplementary crystallographic information

### Comment

As part of our ongoing studies of pyrazole derivatives with potential biological activities (Fun *et al.*, 2011), we have synthesized the title compound, (I), to study its crystal structure.

In the title molecule (Fig. 1), the benzene (C19-C24) ring and the two phenyl (C1-C6 and C13-C18) rings form dihedral angles of 10.34 (16), 50.23 (16) and 40.95 (15)°, respectively, with the pyrazole ring (N1/N2/C10-C12). The benzene ring also forms dihedral angles of 56.89 (17) and 38.81 (16)° with dichloro-bound phenyl (C1-C6) and bromo-bound phenyl (C13-C18) rings, respectively. The phenyl rings form a dihedral angle of 89.57 (17)°. The title molecule exists in *trans* configuration with respect to the acyclic C8═C9 bond [bond length = 1.336 (4) Å]. Bond lengths (Allen *et al.*, 1987) and angles are within normal ranges and are comparable to a related structure (Fun *et al.*, 2011).

In the crystal (Fig. 2), molecules are linked into inversion dimers by pairs of intermolecular C11–H11A···O1 hydrogen bonds (Table 1), generating fourteen-membered D^2^_2_(14) ring motifs (Bernstein *et al.*, 1995). The crystal structure is further consolidated by C15–H15A···Cg1 (Table 1) interactions, where Cg1 is the centroid of C1-C6 phenyl ring.

### Experimental

To a cold, stirred mixture of methanol (20 ml) and sodium hydroxide (12.09 mmol), 2,4-dichloroacetophenone (4.03 mmol) was added. The reaction mixture was stirred for 10 min. 3-(4-Bromophenyl)-1-phenyl-1H-pyrazole-4- carbaldehyde (4.03 mmol) was added to this solution followed by tetrahydrofuran (30 ml). The solution was further stirred for 2 h at 273 K and then at room temperature for 5 h. It was then poured into ice cold water. The resulting solution was neutralized with dil. HCl. The solid that separated out was filtered, washed with water, dried and crystallized from ethanol to yield colourless blocks. Yield: 1.6 g, 80 %. *M.p.*: 457-458 K.

### Refinement

All H atoms were positioned geometrically and refined using a riding model with C–H = 0.93 Å and *U*_iso_(H) = 1.2 *U*_eq_(C).

### Crystal data

**Table:**

C_24_H_15_BrCl_2_N_2_O	*F*(000) = 1000
*M**_r_* = 498.19	*D*_x_ = 1.489 Mg m^−^^3^
Monoclinic, *P*2_1_/*c*	Mo *K*α radiation, λ = 0.71073 Å
Hall symbol: -P 2ybc	Cell parameters from 2554 reflections
*a* = 11.4203 (14) Å	θ = 2.9–22.2°
*b* = 9.9357 (13) Å	µ = 2.11 mm^−^^1^
*c* = 19.656 (3) Å	*T* = 296 K
β = 94.653 (3)°	Block, colourless
*V* = 2222.9 (5) Å^3^	0.38 × 0.21 × 0.11 mm
*Z* = 4	

### Data collection

**Table:**

Bruker SMART APEXII DUO CCD diffractometer	6480 independent reflections
Radiation source: fine-focus sealed tube	2743 reflections with *I* > 2σ(*I*)
graphite	*R*_int_ = 0.056
φ and ω scans	θ_max_ = 30.1°, θ_min_ = 1.8°
Absorption correction: multi-scan (*SADABS*; Bruker, 2009)	*h* = −15→16
*T*_min_ = 0.504, *T*_max_ = 0.803	*k* = −13→13
23842 measured reflections	*l* = −27→22

### Refinement

**Table:**

Refinement on *F*^2^	Primary atom site location: structure-invariant direct methods
Least-squares matrix: full	Secondary atom site location: difference Fourier map
*R*[*F*^2^ > 2σ(*F*^2^)] = 0.052	Hydrogen site location: inferred from neighbouring sites
*wR*(*F*^2^) = 0.140	H-atom parameters constrained
*S* = 0.98	*w* = 1/[σ^2^(*F*_o_^2^) + (0.0493*P*)^2^ + 0.6604*P*] where *P* = (*F*_o_^2^ + 2*F*_c_^2^)/3
6480 reflections	(Δ/σ)_max_ = 0.001
271 parameters	Δρ_max_ = 0.44 e Å^−^^3^
0 restraints	Δρ_min_ = −0.52 e Å^−^^3^

### Special details

**Table:**

Geometry. All esds (except the esd in the dihedral angle between two l.s. planes) are estimated using the full covariance matrix. The cell esds are taken into account individually in the estimation of esds in distances, angles and torsion angles; correlations between esds in cell parameters are only used when they are defined by crystal symmetry. An approximate (isotropic) treatment of cell esds is used for estimating esds involving l.s. planes.
Refinement. Refinement of F^2^ against ALL reflections. The weighted R-factor wR and goodness of fit S are based on F^2^, conventional R-factors R are based on F, with F set to zero for negative F^2^. The threshold expression of F^2^ > 2sigma(F^2^) is used only for calculating R-factors(gt) etc. and is not relevant to the choice of reflections for refinement. R-factors based on F^2^ are statistically about twice as large as those based on F, and R- factors based on ALL data will be even larger.

### Fractional atomic coordinates and isotropic or equivalent isotropic displacement parameters (Å^2^)

**Table:**

	*x*	*y*	*z*	*U*_iso_*/*U*_eq_	
Br1	0.29004 (5)	−0.10153 (6)	0.11448 (2)	0.1353 (3)	
O1	0.12637 (19)	0.64502 (19)	0.42799 (11)	0.0738 (6)	
N1	−0.10263 (19)	0.0347 (2)	0.43425 (11)	0.0521 (5)	
N2	−0.05301 (19)	−0.0315 (2)	0.38311 (10)	0.0532 (6)	
C1	0.2092 (3)	0.4905 (3)	0.28237 (14)	0.0621 (7)	
H1A	0.1332	0.4616	0.2693	0.075*	
C2	0.2907 (3)	0.4908 (4)	0.23478 (16)	0.0844 (10)	
H2A	0.2701	0.4629	0.1903	0.101*	
C3	0.4027 (3)	0.5329 (5)	0.2538 (2)	0.0960 (12)	
C4	0.4336 (3)	0.5741 (4)	0.31980 (19)	0.0865 (11)	
H4A	0.5097	0.6028	0.3325	0.104*	
C5	0.3506 (3)	0.5722 (3)	0.36638 (15)	0.0624 (8)	
C6	0.2362 (2)	0.5316 (3)	0.34922 (13)	0.0509 (6)	
C7	0.1426 (2)	0.5391 (3)	0.39854 (14)	0.0535 (7)	
C8	0.0702 (2)	0.4217 (3)	0.40921 (15)	0.0562 (7)	
H8A	0.0056	0.4330	0.4346	0.067*	
C9	0.0901 (2)	0.2988 (3)	0.38512 (14)	0.0528 (7)	
H9A	0.1547	0.2885	0.3597	0.063*	
C10	0.0198 (2)	0.1802 (3)	0.39515 (14)	0.0510 (6)	
C11	−0.0605 (2)	0.1606 (3)	0.44253 (14)	0.0546 (7)	
H11A	−0.0818	0.2231	0.4745	0.065*	
C12	0.0218 (2)	0.0567 (3)	0.35954 (13)	0.0499 (6)	
C13	0.0901 (2)	0.0206 (3)	0.30199 (13)	0.0513 (6)	
C14	0.1383 (3)	−0.1062 (3)	0.29778 (16)	0.0647 (8)	
H14A	0.1302	−0.1678	0.3327	0.078*	
C15	0.1984 (3)	−0.1429 (3)	0.24257 (18)	0.0773 (9)	
H15A	0.2302	−0.2288	0.2401	0.093*	
C16	0.2106 (3)	−0.0517 (4)	0.19150 (16)	0.0771 (10)	
C17	0.1647 (3)	0.0740 (4)	0.19476 (17)	0.0854 (11)	
H17A	0.1741	0.1356	0.1600	0.102*	
C18	0.1042 (3)	0.1099 (3)	0.24994 (15)	0.0700 (8)	
H18A	0.0724	0.1959	0.2519	0.084*	
C19	−0.1936 (2)	−0.0283 (3)	0.46832 (13)	0.0538 (7)	
C20	−0.2579 (3)	0.0449 (3)	0.51087 (17)	0.0778 (9)	
H20A	−0.2423	0.1359	0.5180	0.093*	
C21	−0.3459 (3)	−0.0174 (4)	0.54300 (19)	0.0943 (12)	
H21A	−0.3899	0.0325	0.5718	0.113*	
C22	−0.3699 (3)	−0.1507 (4)	0.53335 (19)	0.0871 (10)	
H22A	−0.4294	−0.1918	0.5554	0.105*	
C23	−0.3047 (3)	−0.2235 (4)	0.49049 (19)	0.0845 (10)	
H23A	−0.3209	−0.3142	0.4830	0.101*	
C24	−0.2153 (3)	−0.1630 (3)	0.45838 (16)	0.0695 (8)	
H24A	−0.1702	−0.2131	0.4302	0.083*	
Cl1	0.50704 (12)	0.5349 (2)	0.19526 (7)	0.1885 (8)	
Cl2	0.39593 (8)	0.61893 (11)	0.44937 (5)	0.0945 (3)	

### Atomic displacement parameters (Å^2^)

**Table:**

	*U*^11^	*U*^22^	*U*^33^	*U*^12^	*U*^13^	*U*^23^
Br1	0.1461 (4)	0.1639 (5)	0.1070 (4)	−0.0726 (3)	0.0781 (3)	−0.0677 (3)
O1	0.0941 (15)	0.0484 (12)	0.0836 (15)	−0.0100 (11)	0.0363 (12)	−0.0172 (11)
N1	0.0620 (14)	0.0463 (13)	0.0487 (13)	−0.0033 (11)	0.0081 (11)	−0.0024 (10)
N2	0.0659 (14)	0.0474 (13)	0.0470 (12)	−0.0023 (11)	0.0095 (11)	−0.0052 (10)
C1	0.0677 (18)	0.0636 (19)	0.0544 (17)	0.0069 (15)	0.0012 (15)	−0.0050 (14)
C2	0.094 (3)	0.111 (3)	0.0498 (18)	0.027 (2)	0.0131 (18)	−0.0086 (18)
C3	0.077 (3)	0.145 (4)	0.070 (2)	0.030 (2)	0.029 (2)	0.007 (2)
C4	0.061 (2)	0.122 (3)	0.078 (2)	0.0072 (19)	0.0142 (18)	0.008 (2)
C5	0.0647 (19)	0.069 (2)	0.0535 (17)	0.0039 (15)	0.0037 (15)	0.0011 (14)
C6	0.0591 (17)	0.0442 (15)	0.0496 (15)	0.0033 (13)	0.0059 (13)	−0.0035 (12)
C7	0.0624 (17)	0.0453 (16)	0.0533 (16)	−0.0009 (13)	0.0083 (13)	−0.0038 (13)
C8	0.0584 (17)	0.0479 (17)	0.0641 (18)	−0.0018 (13)	0.0156 (14)	−0.0037 (13)
C9	0.0557 (16)	0.0499 (17)	0.0529 (16)	−0.0017 (13)	0.0058 (13)	−0.0014 (13)
C10	0.0548 (16)	0.0441 (16)	0.0539 (16)	−0.0021 (12)	0.0032 (13)	−0.0049 (12)
C11	0.0648 (17)	0.0405 (16)	0.0590 (17)	−0.0009 (13)	0.0086 (14)	−0.0088 (12)
C12	0.0584 (16)	0.0434 (16)	0.0476 (15)	0.0007 (13)	0.0028 (13)	0.0002 (12)
C13	0.0616 (16)	0.0436 (16)	0.0487 (15)	−0.0071 (13)	0.0037 (13)	−0.0040 (12)
C14	0.077 (2)	0.0516 (19)	0.0670 (19)	−0.0017 (15)	0.0178 (16)	0.0001 (14)
C15	0.089 (2)	0.060 (2)	0.087 (2)	−0.0033 (17)	0.034 (2)	−0.0154 (18)
C16	0.083 (2)	0.086 (3)	0.065 (2)	−0.0349 (19)	0.0275 (17)	−0.0257 (18)
C17	0.126 (3)	0.079 (3)	0.0539 (19)	−0.032 (2)	0.021 (2)	0.0017 (17)
C18	0.099 (2)	0.0571 (19)	0.0543 (18)	−0.0068 (17)	0.0096 (17)	0.0015 (14)
C19	0.0604 (17)	0.0539 (17)	0.0463 (15)	−0.0079 (14)	−0.0007 (13)	0.0022 (13)
C20	0.089 (2)	0.065 (2)	0.084 (2)	−0.0081 (17)	0.0355 (19)	−0.0082 (17)
C21	0.093 (3)	0.096 (3)	0.100 (3)	−0.016 (2)	0.045 (2)	−0.012 (2)
C22	0.084 (2)	0.098 (3)	0.082 (2)	−0.030 (2)	0.020 (2)	0.003 (2)
C23	0.093 (2)	0.076 (2)	0.084 (2)	−0.032 (2)	0.006 (2)	−0.0035 (19)
C24	0.084 (2)	0.061 (2)	0.0650 (19)	−0.0174 (17)	0.0129 (17)	−0.0086 (15)
Cl1	0.1164 (9)	0.351 (2)	0.1079 (9)	0.0464 (13)	0.0678 (8)	0.0086 (13)
Cl2	0.0853 (6)	0.1270 (8)	0.0694 (6)	−0.0180 (5)	−0.0044 (5)	−0.0168 (5)

### Geometric parameters (Å, °)

**Table:**

Br1—C16	1.893 (3)	C10—C12	1.414 (4)
O1—C7	1.222 (3)	C11—H11A	0.9300
N1—C11	1.346 (3)	C12—C13	1.469 (4)
N1—N2	1.363 (3)	C13—C18	1.374 (4)
N1—C19	1.425 (3)	C13—C14	1.380 (4)
N2—C12	1.333 (3)	C14—C15	1.379 (4)
C1—C2	1.372 (4)	C14—H14A	0.9300
C1—C6	1.387 (4)	C15—C16	1.368 (5)
C1—H1A	0.9300	C15—H15A	0.9300
C2—C3	1.369 (5)	C16—C17	1.358 (5)
C2—H2A	0.9300	C17—C18	1.380 (4)
C3—C4	1.378 (5)	C17—H17A	0.9300
C3—Cl1	1.723 (3)	C18—H18A	0.9300
C4—C5	1.370 (4)	C19—C20	1.367 (4)
C4—H4A	0.9300	C19—C24	1.373 (4)
C5—C6	1.384 (4)	C20—C21	1.377 (4)
C5—Cl2	1.734 (3)	C20—H20A	0.9300
C6—C7	1.502 (4)	C21—C22	1.363 (5)
C7—C8	1.455 (4)	C21—H21A	0.9300
C8—C9	1.336 (4)	C22—C23	1.374 (5)
C8—H8A	0.9300	C22—H22A	0.9300
C9—C10	1.448 (4)	C23—C24	1.380 (4)
C9—H9A	0.9300	C23—H23A	0.9300
C10—C11	1.373 (4)	C24—H24A	0.9300
			
C11—N1—N2	111.8 (2)	N2—C12—C13	120.2 (2)
C11—N1—C19	128.2 (2)	C10—C12—C13	128.6 (2)
N2—N1—C19	119.8 (2)	C18—C13—C14	118.3 (3)
C12—N2—N1	104.8 (2)	C18—C13—C12	121.1 (3)
C2—C1—C6	122.3 (3)	C14—C13—C12	120.5 (2)
C2—C1—H1A	118.8	C15—C14—C13	121.0 (3)
C6—C1—H1A	118.8	C15—C14—H14A	119.5
C3—C2—C1	119.0 (3)	C13—C14—H14A	119.5
C3—C2—H2A	120.5	C16—C15—C14	119.3 (3)
C1—C2—H2A	120.5	C16—C15—H15A	120.4
C2—C3—C4	120.7 (3)	C14—C15—H15A	120.4
C2—C3—Cl1	120.2 (3)	C17—C16—C15	120.8 (3)
C4—C3—Cl1	119.2 (3)	C17—C16—Br1	119.4 (3)
C5—C4—C3	119.1 (3)	C15—C16—Br1	119.8 (3)
C5—C4—H4A	120.4	C16—C17—C18	119.7 (3)
C3—C4—H4A	120.4	C16—C17—H17A	120.2
C4—C5—C6	122.1 (3)	C18—C17—H17A	120.2
C4—C5—Cl2	117.1 (3)	C13—C18—C17	121.0 (3)
C6—C5—Cl2	120.7 (2)	C13—C18—H18A	119.5
C5—C6—C1	116.7 (3)	C17—C18—H18A	119.5
C5—C6—C7	122.4 (2)	C20—C19—C24	120.4 (3)
C1—C6—C7	120.8 (2)	C20—C19—N1	120.1 (3)
O1—C7—C8	120.8 (3)	C24—C19—N1	119.5 (3)
O1—C7—C6	119.4 (2)	C19—C20—C21	119.3 (3)
C8—C7—C6	119.7 (2)	C19—C20—H20A	120.3
C9—C8—C7	124.5 (3)	C21—C20—H20A	120.3
C9—C8—H8A	117.7	C22—C21—C20	121.3 (3)
C7—C8—H8A	117.7	C22—C21—H21A	119.4
C8—C9—C10	125.7 (3)	C20—C21—H21A	119.4
C8—C9—H9A	117.2	C21—C22—C23	119.0 (3)
C10—C9—H9A	117.2	C21—C22—H22A	120.5
C11—C10—C12	104.6 (2)	C23—C22—H22A	120.5
C11—C10—C9	128.0 (2)	C22—C23—C24	120.6 (3)
C12—C10—C9	127.4 (2)	C22—C23—H23A	119.7
N1—C11—C10	107.6 (2)	C24—C23—H23A	119.7
N1—C11—H11A	126.2	C19—C24—C23	119.4 (3)
C10—C11—H11A	126.2	C19—C24—H24A	120.3
N2—C12—C10	111.2 (2)	C23—C24—H24A	120.3
			
C11—N1—N2—C12	0.1 (3)	C11—C10—C12—N2	0.7 (3)
C19—N1—N2—C12	176.1 (2)	C9—C10—C12—N2	178.7 (2)
C6—C1—C2—C3	0.1 (5)	C11—C10—C12—C13	178.5 (3)
C1—C2—C3—C4	0.1 (6)	C9—C10—C12—C13	−3.5 (4)
C1—C2—C3—Cl1	−179.9 (3)	N2—C12—C13—C18	136.9 (3)
C2—C3—C4—C5	0.1 (6)	C10—C12—C13—C18	−40.7 (4)
Cl1—C3—C4—C5	−179.8 (3)	N2—C12—C13—C14	−40.7 (4)
C3—C4—C5—C6	−0.7 (5)	C10—C12—C13—C14	141.6 (3)
C3—C4—C5—Cl2	177.6 (3)	C18—C13—C14—C15	−0.5 (5)
C4—C5—C6—C1	1.0 (4)	C12—C13—C14—C15	177.3 (3)
Cl2—C5—C6—C1	−177.2 (2)	C13—C14—C15—C16	0.3 (5)
C4—C5—C6—C7	−175.3 (3)	C14—C15—C16—C17	0.2 (5)
Cl2—C5—C6—C7	6.5 (4)	C14—C15—C16—Br1	−178.7 (2)
C2—C1—C6—C5	−0.7 (4)	C15—C16—C17—C18	−0.6 (5)
C2—C1—C6—C7	175.6 (3)	Br1—C16—C17—C18	178.4 (2)
C5—C6—C7—O1	51.9 (4)	C14—C13—C18—C17	0.1 (5)
C1—C6—C7—O1	−124.2 (3)	C12—C13—C18—C17	−177.6 (3)
C5—C6—C7—C8	−129.9 (3)	C16—C17—C18—C13	0.4 (5)
C1—C6—C7—C8	54.0 (4)	C11—N1—C19—C20	6.9 (4)
O1—C7—C8—C9	−172.3 (3)	N2—N1—C19—C20	−168.4 (3)
C6—C7—C8—C9	9.5 (4)	C11—N1—C19—C24	−172.7 (3)
C7—C8—C9—C10	179.8 (3)	N2—N1—C19—C24	12.0 (4)
C8—C9—C10—C11	−16.9 (5)	C24—C19—C20—C21	−0.9 (5)
C8—C9—C10—C12	165.5 (3)	N1—C19—C20—C21	179.6 (3)
N2—N1—C11—C10	0.3 (3)	C19—C20—C21—C22	0.2 (6)
C19—N1—C11—C10	−175.3 (2)	C20—C21—C22—C23	−0.2 (6)
C12—C10—C11—N1	−0.6 (3)	C21—C22—C23—C24	0.8 (6)
C9—C10—C11—N1	−178.6 (2)	C20—C19—C24—C23	1.5 (5)
N1—N2—C12—C10	−0.5 (3)	N1—C19—C24—C23	−178.9 (3)
N1—N2—C12—C13	−178.5 (2)	C22—C23—C24—C19	−1.5 (5)

### Hydrogen-bond geometry (Å, °)

**Table:**

Cg1 is the centroid of C1–C6 phenyl ring.

**Table:**

*D*—H···*A*	*D*—H	H···*A*	*D*···*A*	*D*—H···*A*
C11—H11A···O1^i^	0.93	2.41	3.329 (4)	170
C15—H15A···Cg1^ii^	0.93	2.82	3.666 (3)	152

Symmetry codes: (i) −*x*, −*y*+1, −*z*+1; (ii) *x*, *y*−1, *z*.
